# Effect of Autochthonous Lactic Acid Bacteria-Enhanced Fermentation on the Quality of Suancai

**DOI:** 10.3390/foods11213310

**Published:** 2022-10-22

**Authors:** Xinying Cao, Mingwei Zhao, Sibo Zou, Zhigao Li, Yuzheng Wu, Chaofan Ji, Yingxi Chen, Liang Dong, Sufang Zhang, Huipeng Liang

**Affiliations:** National Engineering Research Center of Seafood, Collaborative Innovation Center of Provincial and Ministerial Co-Construction for Seafood Deep Processing, Liaoning Province Collaborative Innovation Center for Marine Food Deep Processing, School of Food Science and Technology, Dalian Polytechnic University, Dalian 116034, China

**Keywords:** Suancai, lactic acid bacteria, inoculated fermentation, *Lactiplantibacillus plantarum*, *Levilactabacillus brevis*, *Leuconostoc mesenteroides*

## Abstract

The lactic acid bacteria (LABs) used for fermentation have an extremely vital impact on the quality of Suancai, a fermented vegetable. The bacterial diversity and metabolites of inoculated Suancai with LABs, including *Lactiplantibacillus plantarum* (*Lb. plantarum*), *Levilactabacillus brevis* (*Lb. brevis*), and *Leuconostoc mesenteroides* (*Leu. mesenteroides*), were investigated. The inoculation of LABs significantly decreased the pH and the content of nitrite. The Suancai inoculated with LABs had a higher content of the total titratable acidity (TTA) and organic acids than spontaneous fermentation. The LABs inoculation significantly influenced the bacterial community structures, which directly or indirectly caused changes of metabolites. The bacterial community profiles of Suancai inoculated with Lb. plantarum were more similar to spontaneous fermentation. The inoculation of *Lb. plantarum*, *Lb. brevis*, and *Leu. mesenteroides* could increase its abundance in Suancai. Whatever the species inoculated, *Lb. plantarum* was always the predominant bacterium in Suancai after fermentation. The inoculated LABs were positively correlated with most volatile compounds and amino acids. The inoculated LABs significantly improved the volatile compounds and amino acid content of Suancai. This study could contribute to understanding the function of starters in Suancai fermentation and promote the selection of applicable starters for high-quality Suancai production.

## 1. Introduction

Fermented vegetables have a centuries-old history in China, which can be traced back to the Zhou Dynasty. There are a great variety of fermented vegetables in different regions of China; Suancai is a representative fermentation vegetable in northern China [1]. Generally, Suancai is made from cabbages and the manufacture process is putting the cabbages into a jar, adding 2–6% salt and water, and then leaving it for fermentation one month. Suancai is fermented by natural microbial flora which were derived from the materials and the environment. Suancai has become more and more popular due to its peculiar flavor, texture, health functionality, and nutritive value [2].

Many factors could cause immense impacts on the quality of fermented vegetables, such as starters, salt, temperature, and materials. The influence of the materials on the chemical and sensory characteristic of sauerkraut is diverse [3]. Salt and temperature affected the quality characteristic of fermented vegetables through acting on the composition and structure of microflora [4,5,6]. The metabolites during the fermentation primarily come from the metabolic capability of microflora in fermented vegetables. The starters could influence the diversity and structure of microflora during the fermentation process. Therefore, the starters were the critical factor in affecting the quality of Suancai. Then, the selection of starters used for inoculation was crucially important for the production of high-quality Suancai.

Lactic acid bacteria (LAB), including *Lactobacillus*, *Pediococcus*, *Weissella*, *Leuconostoc*, etc., were detected as the dominant microorganisms during the fermentation of fermented vegetables, such as paocai, Suancai, and suansun [1,7,8]. Many starters belonging to LAB were obtained by plate screening and widely used as starters to improve the fermented foods. Many researchers found that LABs were significantly correlated with flavor compounds in fermented vegetables. Xiao et al. reported that *Lactobacillus* was highly associated with 25 flavor compounds and more than 20 flavor compounds were relevant with *Lactobacillus acetotolerans* and *Latilactobacillus sakei* and enhanced the safety of fermented vegetables [9]. Du et al. utilized *Latilactobacillus sakei* to improve vitamin content, increase acidity, and decrease nitrite concentration during sauerkraut fermentation [10]. Song et al. reported that the inoculation of *Lactiplantibacillus plantarum* (*Lb. plantarum*) and *Pediococcus pentosaceus* increased the content of amino acids, alcohols, and aldehydes of Suancai [11]. *Lb. plantarum* was also reported to improve ester aromatic substances of Suancai and leads to a typical component [12]. Similarly, Yang et al. found that inoculated with *Lb. plantarum* had a high abundance of esters, *Leuconostoc mesenteroides* (*Leu. mesenteroides*), and *Weissella cibaria* have an augmented contribution in acids and ketones in northeast sauerkraut [13]. However, Wang et al. found that *Lb. plantarum* inoculation resulted in slow fermentation and massive acid production, while *Leu. mesenteroides* inoculation could accelerate the fermentation, quickly decrease the pungent odor of the raw materials, and improve the flavor and taste of paocai, and *Levilactabacillus brevis* (*Lb. brevis*) inoculation contained ethanol and mannitol [8]. Xiao et al. found that *Enterobacteriaceae* and *Lactobacillaceae* contributed to the synthesis and metabolism of aroma, flavor, and taste compounds during Chinese Sichuan paocai fermentation [14]. Therefore, the species affiliated to LAB had diverse effects on the quality of fermented vegetables. Thus, it is necessary to systematically investigate the effects of LAB-enhanced fermentation on the quality of Suancai.

Therefore, the aim of this study was to determine the effects of different autochthonous *Lb. plantarum*, *Lb. brevis*, and *Leu. mesenteroides* on the bacterial community, physicochemical properties, and flavor quality of Suancai. The results could provide a better understanding of the role of LAB in the Suancai fermentation and a theoretical foundation for the selection of functional starters for the production of high quality Suancai.

## 2. Materials and Methods

### 2.1. Suancai Preparation and Sampling

The fresh cabbages purchased from local market were stripped of damaged leaves, washed, cut in halves, and put into sterilized jars with 6% salt addition (*w/w*). Three LAB strains, namely *Lb. plantarum* (LP), *Lb. brevis* (LB), and *Leu. mesenteroides* (LM), were isolated from Suancai samples and used as the starters with a primary cell density at 10^7^ CFU/mL. The control sample (CON) was prepared as the same manufacturing process without adding starter. All the samples were fermented for 30 days at a constant temperature (15 °C). After fermentation, Suancai samples were collected and stored at −20 °C for the next step of analysis.

### 2.2. Physical and Chemical Analysis

The pH was measured by a pH meter (METTLER TOLEDO FE28, Greifensee, Switzerland). Total titratable acidity (TTA), nitrite concentration of Suancai was determined according to our previous studies [4]. The content of organic acids was determined by HPLC (Agilent 1260 infinity II system, Santa Clara, CA, USA) with a ZORBAX-SB C18 column (5 μm, 4.6 × 250 mm) at 210 nm by using a UV detector (Santa Clara, CA, USA) according to our previous studies [11].

### 2.3. DNA Extraction and High-Throughput Sequencing

A total of 8 mL homogenate samples was used for total DNA extracted. The total DNA of Suancai samples was extracted using MagPure Soil DNA LQ Kit (Angen Biotech, Guangzhou, Guangdong, China) according to the operating manual. The NanoDrop 2000 spectrophotometer (Thermo Fisher Scientific Inc., Waltham, MA, USA) was used to determine the concentration and purity of extracted DNA. The 1.0% agarose electrophoresis were performed at 120 V for 15 min to verify the DNA fragment size. Bacterial 16S rRNA gene was amplified by PCR for barcoded sequencing with primer 27F (5′- AGR GTT YGA TYM TGG CTC AG-3′) and primer 1492R (5′- RGY TAC CTT GTT ACG ACT T-3′) [11]. The PCR was setting as: 98 °C for 30 s, 20 cycles of 98 °C for 15 s, 58 °C for 15 s, 72 °C for 60 s, and 72 °C for 7 min. Sequencing libraries were constructed by blending amplicons according to their concentrations, the sequencing of equal amounts and pair-end were implemented by using the Pacbio Sequel platform by OE Biotech Co. Shanghai, China. The data were deposited in NCBI’s Sequence Read Archive (SRA) with an accession number SRP397129.

### 2.4. Sequence Analysis

The raw tags with average quality less than 20 were filtered and the length longer than 75% length of raw tags were obtained by using Trimmomatic (v0.33, Forschungszentrum Jülich GmbH, Jülich, Germany) [15]. FLASH (v1.2.11, Center for Computational Biology, Baltimore, MD, USA) was used to obtain raw tags with a minimum overlap of 10 base pair (bp) and a maximum error ratio 0.2 which paired-end reads sequencing were divided according to the barcode and assembled [16]. The tags with more than one nucleotide mismatch in primer matching and identified as a putative chimera were discarded by using QIIME software (v1.8.0, Howard Hughes Medical Institute, Boulder, CO, USA) to obtain the effective tags [17]. The valid tags were clustered into operational taxonomic units (OTUs) at a similarity of 97% cutoff by USEARCH (v10.0, Sonoma, CA, USA). To get the classified information, the represented sequences of OTUs were compared against the Silva database by using RDP classifier (version 2.2, Center for Microbial Ecology, East Lansing, MI, USA). Mothur (v1.30, University of Michigan, Ann Arbor, MI, USA) was used to analyze the alpha diversity index of sequences.

### 2.5. GC-MS Analysis

The solid-phase microextraction fiber (DVB/CAR/PDMS, Supelco Inc., Bellefonte, PA, USA) was used to extract the volatiles from the sealed headspace vial (Supelco Inc., Bellefonte, PA, USA) at 60 °C for 20 min and subsequently introduced to a heated GC injection port at 250 °C for 5 min to remove the volatiles. The volatile compounds were determined by GC-MS 7890 A/5977 A (Agilent Technologies Inc., Palo Alto, CA, USA) and the methods completely followed our previous report [18]. A semi-quantitative method was used to calculate the content of volatile compounds by relating the peak areas of volatiles to the peak area of the cyclohexanone internal standard (Aladdin, Los Angeles, CA, USA).

### 2.6. Free Amino Acid Analysis

The content of amino acid was determined by the amino acid analyzer (LA8080, Hitachi High-Technologies Co., Tokyo, Japan). The samples of fermentation brine were filtered through a 0.22 μm water filtration membrane, then mixed with acetone into each tube and placed for 5 min. After centrifugation at 10,000× *g* for 10 min, the supernatant was sucked out to a new EP tube, and then blown dry with nitrogen. After drying, the sample was redissolved in 1 mL HCL (0.02 mol/L), then filtered through 0.22 μm filter membrane and transferred 20 μL into the amino acid analyzer.

### 2.7. E-Tongue Analysis

The e-tongue was detected by a TS-5000Z type taste analysis system (Insent, Atsugi, Japan). It tasted for sour taste, bitter taste, astringency, aftertaste-B, aftertaste-A, delicate taste, richness, and salty taste. Before detection, the sensor was activated for 24 h with an internal solution (3.3 mmol KCl and saturated silver chloride) and a reference solution (30 mmol KCl and 0.3 mmol tartaric acid). The reference electrode was demanded to add the internal solution until the liquid level reached about 5 mm (3.3 mm KCl and saturated silver chloride) and 3.3 mm KCl solution from the top of the glass tube for at least 24 h. After passing the self-test, the Suancai samples were put in a special beaker and the detection method was set.

### 2.8. Statistical Analysis

The chart and statistical analysis were made by Origin v9.0. (OriginLab Corporation, Northampton, MA, USA) The Spearman’s rank correlations were calculated by SPSS v13.0 (SPSS Inc., Chicago, IL, USA). All possible models were created by SIMCA v1.0.1 (Umetrics AB, Umeå, Sweden). The heat map was built using STAMP (v2.1.3, The University of Queensland, Brisbane, Australia) [19].

## 3. Results and Discussion

### 3.1. The Physicochemical Properties in Suancai with Different Starters

**Figure 1:**
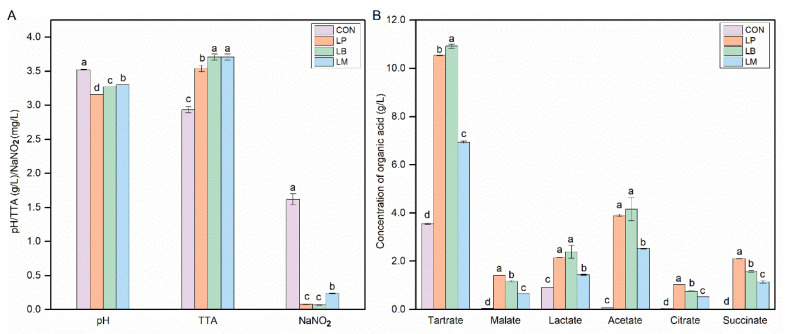
The contents of physicochemical properties (**A**) and organic acids (**B**) in Suancai fermented with different autochthonous LABs. CON, natural fermented Suancai. LP, LB, and LM represent the inoculated Suancai with *Lb. plantarum*, *Lb. brevis*, and *Leu. mesenteroides*. TTA, total titratable acidity, expressed in % lactic acid. Different letters represent significant differences (*p* < 0.05).

The physicochemical properties of Suancai fermented with different starters are shown in Figure 1. The pH and TTA are regarded as important evaluation indexes during the fermentation process of fermented vegetable foods, and the pH values of fermented vegetable were below 4.0 that can be considered the end of fermentation [20]. The inoculation of LABs could significantly decrease the pH and it ranged from 3.16 to 3.52 (Figure 1A). The pH of inoculated Suancai was significantly lower than the CON. LABs can produce organic acids, especially lactic acid. Then, the contents of the TTA and organic acid increased significantly in the inoculated Suancai with LABs in this study (Figure 1A,B). As a homofermentative LAB, *Lb. plantarum* could produce many acids, especially lactic acid. Therefore, the inoculation of *Lb. plantarum* had the lowest pH (Figure 1A) and a high content of lactate (Figure 1B). In addition, the contents of malate, citrate, and succinate in the LP were the highest (Figure 1B). *Lb. brevis*, an obligately heterofermentative LAB, produced a mixture of lactic acid, acetic acid, ethanol, and CO_2_, and then its inoculation obtained a high content of lactate and acetate (Figure 1B). However, the inoculation of *Leu. Mesenteroides*, as also a heterofermentative LAB, had a lower content of lactate and acetate than the inoculation of *Lb. brevis*. Potentially, *Leu. Mesenteroides* was an acid-sensitive microorganism and commonly grow well with an initial pH [21]. This is in accordance with previous studies on paocai [22].

LAB had the nitrite degradation ability and its inoculation could decrease the nitrite concentration in fermented food [10,13,23]. Compared with the CON, the inoculation of the LP, LB, and LM could significantly decrease the nitrite concentration of Suancai in this study (Figure 1A). Three groups had different nitrite depletion abilities, LB and LP had a better capability to reduce nitrite compared with LM. Nitrite depletion is important for the safety of Suancai and the nitrite concentration was below the GB standard after fermentation (Figure 1A).

### 3.2. The Diversity of Microbial Community in Suancai with Different Starters

Based on the sequencing, 83,933 16S rRNA gene clean tags were obtained from 12 Suancai samples. By means of assembling and filtering, about 81,958 valid tags remained for the next study. The valid tags of all samples ranged from 1480.27 to 1487.86 bp. The coverage of samples was more than 0.99 (Table 1), which showed that the main bacterial phylotypes of samples had been covered. After removing the chimera processes, the bacteria numbers of observed species were 16.00–28.57 (Table 1). The assessment of the species number was evaluated by the Chao1 indices. The Chao1 values of LP, LB, and LM were higher than that of CON (Table 1). The Shannon and Simpson indices as diversity estimators, represented the approximated number of OTUs and the evenness of their distribution in the samples. The samples fermented with different starters had significantly higher values of Shannon and Simpson indices than the CON (Table 1). After fermentation, the LM had the highest Simpson and Shannon index of bacterial communities.

### 3.3. The Composition of the Bacterial Community in Suancai with Different Starters

Microorganisms are the most important factor in the quality of fermented foods. High-throughput sequencing has been widely applied to reveal the microbial community in fermented food [4,5,9,13]. In this study, the bacterial community at the phylum, genus, and species were revealed by high-throughput sequencing in Suancai (Figure S1). The number of OTUs taxonomy reached the saturation plateau, indicating that the species in this environment will not increase significantly with the increase of sample size, which means that the sampling is sufficient (Figure S1A). Firmicutes was discovered as the dominant phylum and accounted for more than 80% in all Suancai samples (Figure S1B). Firmicutes were always observed as the dominant phylum in fermented vegetables [1,2,24]. The second most dominant phylum was Proteobacteria. At the phylum level, no remarkable differences were found in bacterial abundance. At the genus level, *Lactobacillus* was the dominant bacterium in all samples, which always predominated in other fermented vegetables foods [7,25]. Therefore, many species belonging to *Lactobacillus* were used to improve the quality of fermented vegetables [10,11,12]. Some species of *Lactobacillus* could perform homolactic or heterolactic fermentation to produce lactic acid, acetic acid, ethanol, etc., which can explain the high sensory quality of fermented vegetables because these metabolites had an active influence on the improvement of sensory characteristics [26].

**Figure 2:**
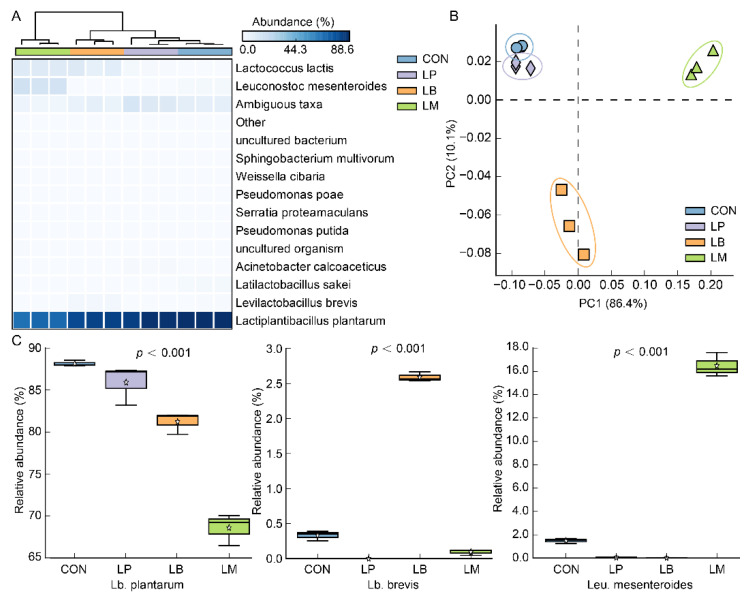
The heatmap (**A**), PCA (**B**), and relative abundance (**C**) of the bacterial community at the species level in Suancai fermented with different autochthonous LABs.

At the species level, a total of 35 species were discovered in all samples and *Lb*. *plantarum* was detected as the dominant species in Suancai (Figure 2A). Possibly, *Lb. plantarum* is a widely distributed LAB and could tolerate low pH [27,28], indicating that it can survive in the late fermentation stage of Suancai. Therefore, whichever species of LAB were inoculated, *Lb. plantarum* was always the dominant bacterium after fermentation of Suancai (Figure 2A). As a kind of homofermentative LAB, *Lb. plantarum* was always observed as the dominant species in fermented vegetables [24,29] and was frequently used in the fermentation of vegetables and fruit juices to improve the quality of products [30,31]. The results of principal components analysis (PCA) based on the bacterial community are shown in Figure 2B and the variation of two canonical axes explained 10.1% and 86.4%. The inoculated fermentation of starters affected the bacterial community structures of Suancai (Figure 2B). Compared with LB and LM, the bacterial community profiles of the LP were more similar to that of CON (Figure 2B), which may be on account of the *Lb. plantarum* being observed as the dominant bacterium after fermentation of Suancai. On account of the addition of starters, *Lb. brevis* and *Leu. mesenteroides* were observed with high abundance in LB and LM, respectively (Figure 2A,C). Moreover, *Lactococcus lactis* were observed with a large abundance in LB and LM (Figure 2A) on account of the slower acidification than LP. As one of the first bacteria to occupy plant material, *Lactococcus lactis* had a weak acid resistance and became the less dominant species as the pH lowered [32].

### 3.4. Volatile Compound Analysis in Suancai with Different Starters

**Figure 3:**
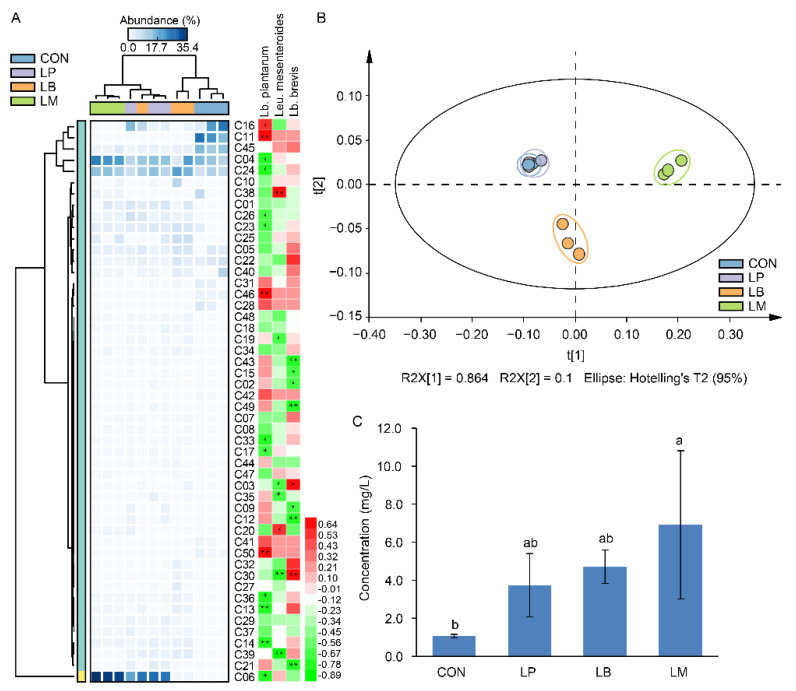
The heatmap and correlation analysis with LABs (**A**), scatter plot (**B**), and total concentration (**C**) of volatile flavor compounds in Suancai fermented with different autochthonous LABs. Different letters represent significant differences (*p* < 0.05). * *p* < 0.05, ** *p* < 0.01.

A total of 50 volatile compounds were found in Suancai samples with different LABs (Supplementary Table S1 and Figure 3A). *Lb. plantarum*, *Lb. brevis*, and *Leu. mesenteroides* were positively correlated to 16, 28, and 14 volatile compounds (Figure 3A). The LAB had proteolytic activity and its inoculation affected the microbial community structures [11,13], and then indirectly or indirectly affected the profiles of volatile compounds in Suancai (Figure 3B). The results of PCA showed that the volatile compounds in Suancai of LB and LM were clustered as a separate group, the group of CON and LP had a higher resemblance, indicating that they had similar profiles of volatile compound (Figure 3B). This might be due to their parallel bacterial community (Figure 2B), which were correlated with flavors in fermented vegetables [5,9,24]. In the previous study, *Lb. plantarum*-inoculated fermentation contributed to the increase of esters, while inoculated *Leu. mesenteroides* accumulated acids and ketones [13]. However, in this study, the inoculation of *Lb. plantarum*, *Leu. Mesenteroides*, and *Lb. brevis* mainly increased the contents of alcohols, such as 3-hexen-1-ol, 1-hexanol, phenylethyl alcohol, 1-octanol, etc. (Supplementary Table S1 and Figure 3A). Potentially, the differences in the strains were responsible for this disagree. In a previous study on paocai, the inoculation of LP, LB, and LM had significant effects on the alcohols, which was similar to our results [22]. In addition, the group of LP had a high concentration of alcohols, which was similar to a previous study that inoculation with *Lb. plantarum* promoted the production of some alcohols [33]. Except the alcohols, the *Leu. mesenteroides* inoculation also increased the content of isothiocyanates, such as 4-isothiocyanato-1-butene and (2-isothiocyanatoethyl)-benzene, which were produced by the hydrolysis of glucosinolates and had a bitter taste [34]. Moreover, aldehydes also distinguish LB from the other inoculation groups, and the main element is benzaldehyde, it provides an almond flavor and a fruity aroma [35]. In total, the inoculation of LABs increased the total concentration of volatile compounds in Suancai (Figure 3C). In fermented foods, volatile compounds were associated with the microflora during the fermentation [2,5,7]. Thus, the higher diversity of microbial community (Table 1) accounting for the LM had the highest content of volatile compounds, followed by LB. Therefore, the effects of inoculated LABs on the volatile compounds were diverse and depended on the species of starters used for fermentation.

### 3.5. Changes of the Free Amino Acid in Suancai with Different Starters

**Figure 4:**
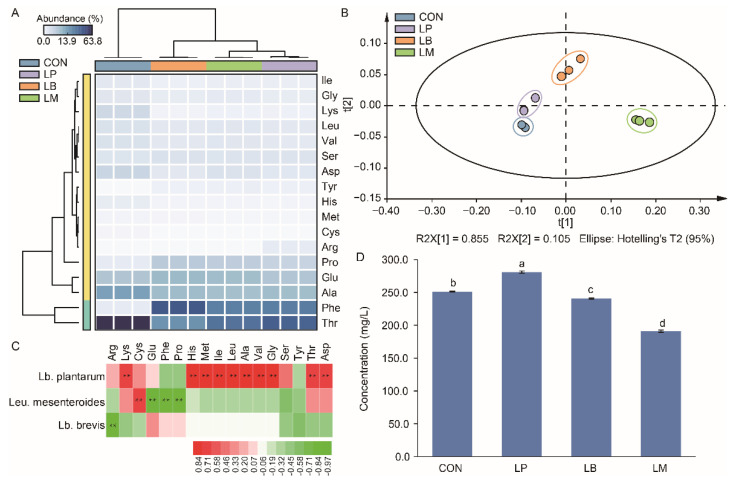
The heatmap (**A**), scatter plot (**B**), correlation analysis with LABs (**C**), and total concentration (**D**) of amino acid in Suancai fermented with different autochthonous LABs. Different letters represent significant differences (*p* < 0.05). ** *p* < 0.01.

The results of the free amino acid contents in Suancai are displayed in Supplementary Table S1 and there were 17 free amino acids were detected in this study. According to the heatmap, the content of threonine acid (Thr), phenylalanine acid (Phe), alanine acid (Ala), glutamic acid (Glu), and proline acid (Pro) were the main free amino acids in Suancai (Figure 4A). The PLS analysis showed the influence of different strains on the amino acid profiles of Suancai (Figure 4B). The Suancai samples were gathered into different group according to the PCA analysis, indicating that LAB inoculation could affect the profiles of amino acids in Suancai. *Lb. plantarum*, *Leu. Mesenteroides*, and *Lb. brevis* were significantly correlated to the amino acids (Figure 4C). *Lb. plantarum* was significantly positive correlated with 10 amino acids. Therefore, the inoculation of *Lb. plantarum* had the highest content of free amino acids, while LM had the lowest content of amino acids (Figure 4D). *Lb. plantarum* was reported to generate protein degradation and proteolytic enzymes to produce amino acids [36]. Therefore, the inoculation of *Lb. plantarum* increased the content of amino acids in Suancai in this study (Supplementary Table S2), which was also observed in previous studies [11,13]. Amino acid, as the precursor of alcohols, aldehydes, esters, and ketonic acids, participated indirectly in flavor development and contributed directly to the taste of Suancai [27,37]. As branched-chain amino acids, isoleucine (Ile), leucine (Leu), and valine (Val) can be converted to α-keto acids by LAB, and then translated into aldehydes and alcohols [38]. Consequently, the contents of Ile, Leu, and Val in CON were lower than that in inoculated group (Figure 4A). Potentially, the LAB increased the content of volatile compounds in Suancai through the converting of the amino acid. LM had the lowest content of amino acid and the highest content of volatile compounds (Figure 3C and 4D). Therefore, the inoculation of *Leu. mesenteroides* was more beneficial to the convert of amino acid to volatile compounds on account of its slow acidification process obtained a high bacterial diversity.

**Figure 5:**
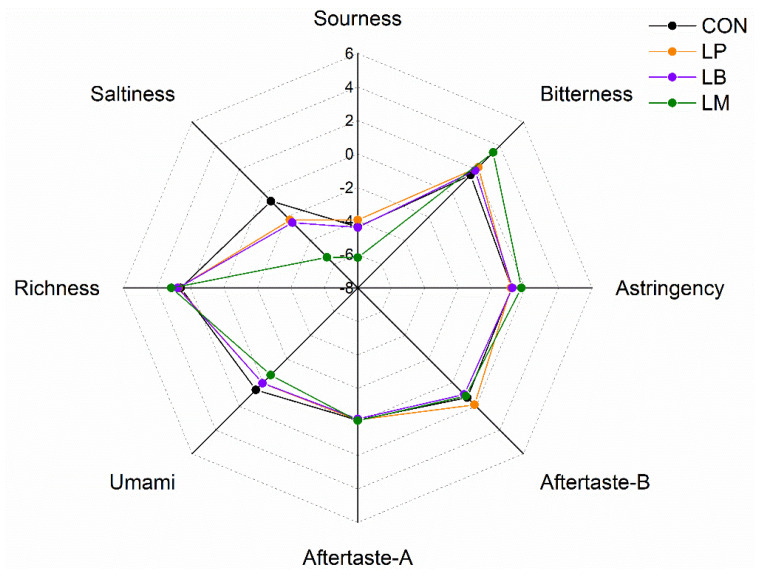
The E-tongue profiles of Suancai fermented with different autochthonous LABs.

Moreover, amino acids contributed to the taste of Suancai. The results of electronic tongue data of Suancai with LABs were displayed in (Figure 5). The taste profiles of Suancai were different with different LABs. After fermentation, Thr was the richest amino acid (Figure 4A). As it is a sweet amino acid [39], Thr could improve the taste of Suancai. LM had the lowest saltiness, sourness, and umami, but had the highest bitterness and astringency (Figure 5), which were an awful taste for Suancai. The sourness, bitterness, astringency, aftertaste-B, aftertaste-A, and richness of the LP and LB had no significant difference. The CON had the highest Asp content. Aspartic acid (Asp) and glutamic acid (Glu) were relevant to umami flavor which considered the basic taste in cooking [40]. Potentially, © CON had the highest umami taste on account of its high Asp content. These results indicated that LABs could change the FAAs composition to upgrade the taste of Suancai.

## 4. Conclusions

Three strains of *Lb. plantarum*, *Lb. brevis*, and *Leu. Mesenteroides* were used as starters for Suancai fermentation. The LAB inoculation significantly decreased the pH and the content of nitrite. The Suancai inoculated with LABs had a higher content of the TTA and organic acids than spontaneous fermentation. According to the PCA analysis, the LABs inoculation significantly influenced the bacterial community of Suancai. The inoculation could increase the LAB abundance in Suancai. The bacterial community profiles of Suancai inoculated with *Lb. plantarum* was more similar to spontaneous fermentation. No matter what species were inoculated, *Lb. plantarum* was always the dominant bacterium after fermentation. The concentration of volatile compound in inoculated Suancai was higher than the CON. The inoculated LABs were positively correlated with most volatile compounds and amino acids. The inoculation of LAB could affect the bacterial profiles of Suancai which directly or indirectly caused the changes of organic acids and metabolites. This study could contribute to select the applicable and functional starters for Suancai fermentation.

## Figures and Tables

**Table 1:** Alpha index of Suancai fermented with different autochthonous LABs.

Samples ^1^	CON	LP	LB	LM
Chao1	20.61 ± 5.29 ^a^	34.60 ± 2.45 ^a^	29.38 ± 4.20 ^a^	31.70 ± 13.17 ^a^
Goods coverage	0.99 ± 0.00 ^a^	0.99 ± 0.00 ^a^	0.99 ± 0.00 ^a^	0.99 ± 0.00 ^a^
Shannon	0.75 ± 0.03 ^c^	1.00 ± 0.11 ^b^	1.11 ± 0.04 ^b^	1.48 ± 0.09 ^a^
Observed species	16.00 ± 2.33 ^b^	28.57 ± 0.76 ^a^	24.23 ± 3.48 ^ab^	25.93 ± 6.05 ^a^
Simpson	0.22 ± 0.01 ^c^	0.26 ± 0.03 ^c^	0.33 ± 0.01 ^b^	0.49 ± 0.02 ^a^
PD whole tree	1.39 ± 0.14 ^b^	2.56 ± 0.09 ^a^	2.14 ± 0.26 ^ab^	2.57 ± 0.68 ^a^

^1^ CON, natural fermented Suancai. LP, LB, and LM represent the inoculated Suancai with *Lb. plantarum*, *Lb. brevis*, and *Leu. Mesenteroides*. Different superscript letters in a row indicate significant differences (*p* < 0.05).

## Data Availability

The data presented in this study are available on request from the corresponding author.

## Supplementary Materials

The following supporting information can be downloaded at: https://www.mdpi.com/article/10.3390/foods11213310/s1, Figure S1: The species accumulation curves (A), relative abundance of bacterial communities at phylum (B), genus (C), and species (D) level in Suancai fermented with different autochthonous LABs. Table S1: The concentration of volatile flavor compounds in Suancai fermented with different LABs. Table S2: The concentration of free amino acid in Suancai fermented with different autochthonous LABs.
