# Supplementary material for: Effect of Autochthonous Lactic Acid Bacteria-Enhanced Fermentation on the Quality of Suancai
Source: Foods. 2022 Oct 22;11(21):3310. doi: 10.3390/foods11213310 (PMC9657813; doi:10.3390/foods11213310)
Supplement: Supplementary file 1 [file foods-11-03310-s001.zip › Supplementary Figure S1.pdf]

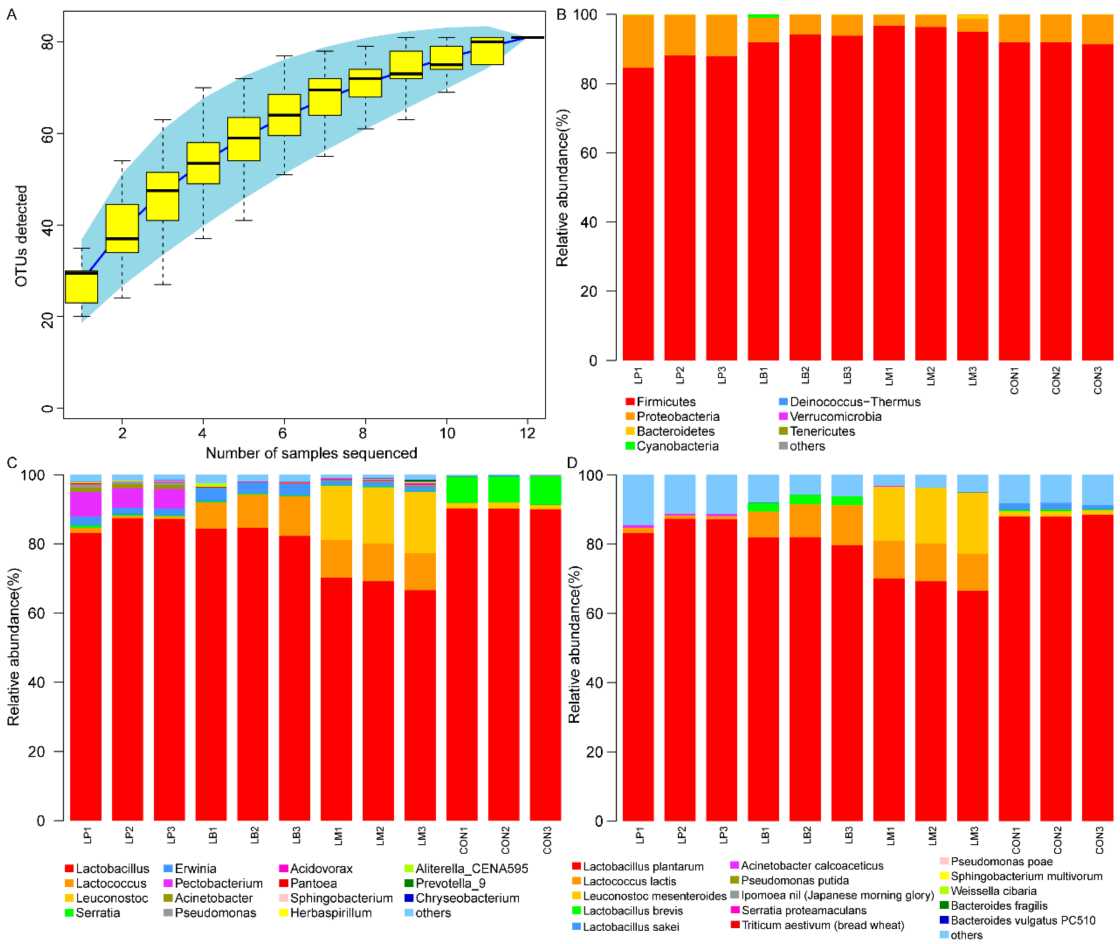

Figure S1 The species accumulation curves (A), relative abundance of bacterial communities at phylum (B), genus (C) and species (D) level in Suancai fermented with different autochthonous LABs. CON, natural fermented Suancai. LP, LB and LM represent the inoculated Suancai with *Lb. plantarum*, *Lb. brevis* and *Leu. mesenteroides*.
