# Supplementary material for: Effect of Autochthonous Lactic Acid Bacteria-Enhanced Fermentation on the Quality of Suancai
Source: Foods. 2022 Oct 22;11(21):3310. doi: 10.3390/foods11213310 (PMC9657813; doi:10.3390/foods11213310)
Supplement: Supplementary file 1 [file foods-11-03310-s001.zip › Supplementary Table S1.pdf]

Table S1 The concentration of volatile flavor compounds in Suancai fermented with different LABs

NO.	Compounds	RI	Formula	CON	LP	LB	LM
C01	Acetic acid	1453	$C_2H_4O_2$	nd	0.202±0.101^{a}	0.148±0.062^{a}	0.124±0.134^{a}
C02	Dimethyl trisulfide	1344	$C_2H_6S_3$	nd	0.008±0.005	nd	nd
C03	1,1-Dicyanoethane	1237	$C_4H_4N_2$	nd	nd	0.029±0.025	nd
C04	4-Isothiocyanato-1-Butene	1433	C_5H_7NS	0.140±0.014^{b}	0.469±0.191^{ab}	0.556±0.220^{ab}	1.554±0.907^{a}
C05	5-Cyano-1-pentene	1332	C_6H_9N	0.033±0.023^{b}	0.097±0.048^{ab}	0.256±0.073^{a}	0.189±0.103^{ab}
C06	1-Cyano-4,5-epithiopentane	2079	C_6H_9NS	nd	0.847±0.337^{a}	0.396±0.498^{a}	2.352±1.337^{a}
C07	3-Hexen-1-ol	1383	$C_6H_{12}O$	nd	0.010±0.007^{a}	0.021±0.003^{a}	0.016±0.023^{a}
C08	1-Hexanol	1354	$C_6H_{14}O$	nd	0.012±0.009^{a}	0.014±0.010^{a}	0.010±0.010^{a}
C09	Benzonitrile	1584	C_7H_5N	nd	0.064±0.046	nd	nd
C10	Benzaldehyde	1307	C_7H_6O	nd	0.004±0.006^{a}	0.199±0.281^{a}	0.047±0.066^{a}
C11	Butanedioic acid, methylene-, dimethyl ester	1097	$C_7H_{10}O_4$	0.223±0.034	nd	nd	nd
C12	8-Oxabicyclo[5.1.0]octane	1677	$C_7H_{12}O$	nd	0.061±0.042	nd	nd
C13	6-(Methylthio)hexanenitrile	2052	$C_7H_{13}NS$	nd	nd	0.105±0.042^{a}	0.067±0.038^{a}
C14	Berteroin	2263	$C_7H_{13}NS_2$	nd	0.063±0.016^{a}	0.113±.028^{a}	0.132±0.080^{a}
C15	1-Heptanol	1448	$C_7H_{16}O$	nd	0.011±0.008	nd	nd
C16	2-Amino-4-methyl-Benzoic acid	949	$C_8H_9NO_2$	0.183±0.110^{a}	0.333±0.352^{a}	0.158±0.172^{a}	nd
C17	Phenylethyl Alcohol	1698	$C_8H_{10}O$	nd	0.012±0.011^{a}	0.019±0.017^{a}	0.022±0.014^{a}

C18	(E)-2-Octenal	1476	$C_8H_{14}O$	nd	0.044±0.034[a]	0.036±0.015[a]	0.028±0.015[a]
C19	1-Octen-3-ol	1442	$C_8H_{16}O$	nd	0.032±0.023[a]	0.036±0.027[a]	nd
C20	(E)-2-Octen-1-ol	1599	$C_8H_{16}O$	nd	0.045±0.064[a]	nd	0.074±0.038[a]
C21	Octanoic acid	1852	$C_8H_{16}O_2$	nd	0.109±0.040	nd	nd
C22	1-Octanol	1545	$C_8H_{18}O$	0.053±0.017[b]	0.067±0.035[b]	0.199±0.087[a]	0.081±0.045[ab]
C23	Benzenepropanenitrile	2032	C_9H_9N	0.011±0.016[b]	0.149±0.060[ab]	0.254±0.094[a]	0.206±0.114[a]
C24	(2-isothiocyanatoethyl)-Benzene	2225	C_9H_9NS	0.098±0.020[a]	0.362±0.112[a]	0.789±0.295[a]	0.933±0.527[a]
C25	2,4-dimethyl-Benzaldehyde	1797	$C_9H_{10}O$	nd	0.066±0.093[a]	0.259±0.195[a]	0.306±0.221[a]
C26	Isophorone	1575	$C_9H_{14}O$	nd	0.179±0.144[a]	0.151±0.046[a]	0.119±0.065[a]
C27	3,5,5-trimethyl-2-Hexene	1276	C_9H_{18}	nd	0.002±0.002[a]	0.078±0.110[a]	nd
C28	(6Z)-Nonen-1-ol	1171	$C_9H_{18}O$	0.029±0.022	nd	nd	nd
C29	1-Nonanol	1647	$C_9H_{20}O$	nd	0.081±0.049[a]	0.049±0.021[a]	0.037±0.018[a]
C30	Naphthalene	1716	$C_{10}H_8$	nd	nd	0.057±0.041	nd
C31	4-propyl-Benzaldehyde	1617	$C_{10}H_{12}O$	0.021±0.016[a]	0.002±0.003[a]	0.017±0.025[a]	nd
C32	1,2,4,5-tetramethyl-Benzene	1404	$C_{10}H_{14}$	nd	nd	0.062±0.073	nd
C33	3-Methylene-1-oxa-spiro[4.5]decan-2-one	1995	$C_{10}H_{14}O_2$	nd	0.016±0.007[a]	0.027±0.007[a]	0.021±0.011[a]
C34	3,7-dimethyl-1,3,7-Octatriene	1026	$C_{10}H_{16}$	nd	0.019±0.004[a]	0.051±0.027[a]	0.021±0.018[a]
C35	2,6-dimethyl-2,4,6-Octatriene	1339	$C_{10}H_{16}$	nd	0.022±0.010[a]	0.011±0.008[a]	nd
C36	β-Myrcene	1143	$C_{10}H_{16}$	nd	0.033±0.016[a]	0.085±0.044[a]	0.071±0.042[a]

C37	trans, trans-Octa-2,4-dienyl acetate	1671	$C_{10}H_{16}O_2$	nd	0.075±0.050[a]	0.049±0.016[a]	0.060±0.030[a]
C38	(Z)- 3,7-dimethyl-2,6-Octadien-1-ol	1846	$C_{10}H_{18}O$	0.031±0.044[a]	nd	nd	0.102±0.061[a]
C39	Geraniol	1845	$C_{10}H_{18}O$	nd	0.070±0.026[b]	0.153±0.048[a]	nd
C40	Linalool	1338	$C_{10}H_{18}O$	0.043±0.061[ab]	0.008±0.011[b]	0.132±0.059[a]	0.082±0.047[ab]
C41	Citronellol	1228	$C_{10}H_{20}O$	0.007±0.005	nd	nd	nd
C42	Decanal	1206	$C_{10}H_{20}O$	0.003±0.002	nd	nd	nd
C43	Dodecanoic acid	2271	$C_{12}H_{24}O_2$	nd	0.011±0.004	nd	nd
C44	1-Dodecanol	1763	$C_{12}H_{26}O$	nd	0.028±0.012[a]	0.019±0.026[a]	nd
C45	2,4-Di-tert-butylphenol	2297	$C_{14}H_{22}O$	0.153±0.020[a]	0.062±0.017[a]	0.131±0.028[a]	0.224±0.170[a]
C46	2,6-Di-Tert-Butyl-4-Methylphenol	1513	$C_{15}H_{24}O$	0.022±0.002	nd	nd	nd
C47	4,6-di-tert-Butyl-m-cresol	2090	$C_{15}H_{24}O$	nd	nd	0.015±0.021[a]	0.016±0.016[a]
C48	Butylated Hydroxytoluene	1898	$C_{15}H_{24}O$	nd	0.030±0.014[a]	0.037±0.024[a]	0.018±0.020[a]
C49	n-Hexadecanoic acid	2697	$C_{16}H_{32}O_2$	nd	0.031±0.010	nd	nd
C50	Heptadecane	1700	$C_{17}H_{36}$	0.014±0.004	nd	nd	nd
	Total			1.065±0.096[b]	3.739±1.666[ab]	4.710±0.878[ab]	6.913±3.899[a]

The concentrations were calculated through an internal standard method (mg/kg)

Values are means ± standard deviation. Different superscript letters in a row indicate significant differences ($p < 0.05$).

nd, not detected.

CON, natural fermented Suancai. LP, LB and LM represent the inoculated Suancai with *Lb. plantarum*, *Lb. brevis* and *Leu. mesenteroides*.
