# Supplementary material for: Effect of Autochthonous Lactic Acid Bacteria-Enhanced Fermentation on the Quality of Suancai
Source: Foods. 2022 Oct 22;11(21):3310. doi: 10.3390/foods11213310 (PMC9657813; doi:10.3390/foods11213310)
Supplement: Supplementary file 1 [file foods-11-03310-s001.zip › Supplementary Table S2.pdf]

Table S2 The concentration of free amino acid in Suancai fermented with different autochthonous LABs.

Samples	CON	LP	LB	LM
Asp	17.19 ± 0.09^{a}	8.62 ± 0.03^{b}	7.19 ± 0.02^{d}	7.79 ± 0.08^{c}
Thr	84.68 ± 0.31^{a}	73.84 ± 0.73^{b}	44.80 ± 0.28^{c}	45.15 ± 0.25^{c}
Ser	9.82 ± 0.06^{b}	10.50 ± 0.02^{a}	9.80 ± 0.08^{b}	7.30 ± 0.07^{c}
Glu	26.11 ± 0.04^{c}	28.65 ± 0.08^{b}	30.20 ± 0.14^{a}	22.86 ± 0.26^{d}
Pro	5.76 ± 0.02^{d}	20.67 ± 0.30^{b}	21.87 ± 0.29^{a}	16.77 ± 0.16^{c}
Gly	8.12 ± 0.02^{a}	6.03 ± 0.02^{b}	5.23 ± 0.03^{c}	4.77 ± 0.05^{d}
Ala	41.86 ± 0.09^{a}	33.69 ± 0.11^{b}	29.39 ± 0.16^{c}	21.92 ± 0.26^{d}
Cys	0.91 ± 0.01^{a}	0.07 ± 0.00^{b}	nd	0.07 ± 0.00^{c}
Val	10.91 ± 0.01^{a}	7.75 ± 0.02^{b}	6.88 ± 0.02^{c}	6.27 ± 0.07^{d}
Met	1.51 ± 0.00^{a}	1.13 ± 0.01^{b}	0.89 ± 0.02^{c}	0.83 ± 0.01^{d}
lle	7.27 ± 0.01^{a}	4.53 ± 0.01^{b}	3.67 ± 0.02^{c}	3.38 ± 0.04^{d}
Leu	10.72 ± 0.02^{a}	8.18 ± 0.30^{b}	7.05 ± 0.02^{c}	6.31 ± 0.10^{d}
Tyr	0.30 ± 0.00^{c}	4.07 ± 0.47^{a}	3.16 ± 0.01^{b}	3.04 ± 0.19^{b}
Phe	5.88 ± 0.05^{d}	61.76 ± 0.91^{b}	67.05 ± 0.33^{a}	41.12 ± 0.40^{c}
Lys	15.55 ± 0.00^{a}	3.34 ± 0.24^{b}	1.94 ± 0.00^{d}	2.31 ± 0.02^{c}
His	5.55 ± 0.02^{a}	1.53 ± 0.06^{b}	1.38 ± 0.00^{c}	1.13 ± 0.01^{d}
Arg	nd	6.45 ± 0.04^{a}	nd	nd
Total	251.14 ± 0.68^{b}	280.81 ± 1.71^{a}	240.50 ± 0.99^{c}	191.02 ± 1.67^{d}

Values are means ± standard deviation and are expressed as mg/L. Different superscript letters in a row indicate significant differences ($p < 0.05$). CON, natural fermented Suancai. LP, LB and LM represent the inoculated Suancai with *Lb. plantarum*, *Lb. brevis* and *Leu. mesenteroides*.
